# A Case of Dislocation of the Ulna Backwards and the Radius Forwards

**Published:** 1886-03

**Authors:** R. J. H. Scott

**Affiliations:** Assistant Surgeon, Royal United Hospital, Bath.


### Clinical Records.

### A CASE OF DISLOCATION OF THE ULNA BACKWARDS AND THE RADIUS FORWARDS. By R. J. H. Scott, M.R.C.S. Eng., Assistant Surgeon, Royal United Hospital, Bath.

F. J. D., aged 46, came to the hospital about an hour and a half after meeting with the above accident. He was riding a horse, "to show it off," when he was thrown over the horse's head, and fell on his hands and head. He found his left elbow dislocated, and the forearm was in a semi-flexed position. He asked a gentleman to pull the arm "straight," as he thought that was the best position for the arm to be in. The gentleman did so, and F. J. D. felt "something" give way in the joint, and a bone appeared to slip forward.

On admission, the left arm was almost straight, the elbow much swollen, and he could move it only very slightly. The prominences of the external and internal condyles of the humerus had disappeared; the olecranon process of the ulna was not in a line with the condyles, but about one and a half inch above them posteriorly. On the patient extending his forearm to the full, a sulcus appeared on each side of the tendon of the triceps at its attachment to the olecranon. The head of the radius could not be felt behind the elbow.

The dislocated ulna was easily reduced by the knee being placed in the bend of the elbow; but the forearm could not be fully flexed. The head of the radius could now easily be felt resting on the anterior surface of the external condyle of the humerus, and could be made to slightly rotate there. I then, with my knee in the bend of the elbow, pulled on the forearm by the hand, and gradually continued to flex the forearm, while the House Surgeon pressed the head of the radius into position with his thumbs. I was now able to completely flex the forearm, and the head of the radius could be felt to be in good position, though apparently more prominent anteriorly than normal. The arm was placed on an angular splint, the elbow being left exposed for the application of evaporating lotions. The arm was kept on the splint about a week, and in a sling for a fortnight. Eight weeks after the accident, the patient is able to use his left arm fairly well. He can rotate the left forearm as well as his right; his power of extension is almost complete, but he cannot flex the forearm sufficient to touch his shoulder with the fingers, and he is not yet able to strike out with the left arm as well as his right. The prominences of bone of the elbow-joint are all in normal position; but, on extending the forearm, there is still some fulness on each side of the tendon of the triceps at its attachment to the olecranon. I have advised him to use dumb-bells of moderate weight, and to carry weights with his left hand.

*Remarks.*—The above case is interesting on account of the rarity of the accident,—Erichsen mentions "that he has only seen two cases in hospital practice,"—and also on account of the manner in which it was produced, the usual cause being a fall on the hand, with a wrench of the limb at the same time.

